# Subacromial Balloon Spacer Versus Partial Rotator Cuff Repair in the Treatment of Massive Irreparable Rotator Cuff Tears: Facility Personnel Allocation and Procedural Cost Analysis

**DOI:** 10.7759/cureus.41538

**Published:** 2023-07-07

**Authors:** Tyler A Luthringer, Mohamad Y Fares, Alexander J Rondon, Alayna K Vaughan, Adam Z Khan, Joseph A Abboud

**Affiliations:** 1 Division of Hand and Upper Extremity, Midwest Orthopaedics at Rush, Rush University, Chicago, USA; 2 Division of Shoulder and Elbow Surgery, Rothman Orthopaedic Institute, Thomas Jefferson University, Philadelphia, USA

**Keywords:** cost, arthroscopic repair, rotator cuff tear, shoulder, balloon arthroplasty

## Abstract

Background

The subacromial balloon is a novel technology that has shown promise in managing a select patient population with massive irreparable rotator cuff tears. The purpose of this study was to quantify the true facility cost difference between subacromial balloon placement (SBP) and partial rotator cuff repair (PCR).

Methodology

A prospective cohort of patients with massive irreparable rotator cuff tears randomized to SBP versus PCR between 2015 and 2018 was retrospectively reviewed. Demographic variables, medical comorbidities, and range-of-motion (ROM) outcomes for all patients were recorded. True facility costs with respect to personnel were calculated using a time-driven activity based-costing (TDABC) algorithm and were classified into personnel costs and supply costs.

Results

Seven patients were treated with PCR compared to nine treated with SBP. No significant differences were observed with respect to demographic characteristics. Postoperative mean external rotation was 37° in SBP patients significantly higher than that of PCR patients at 8° (*P *= 0.023). Personnel time and cost differences while in the operating room (OR) were significantly less for the SBP ($605.58) compared to PCR ($1362.76) (*P *< 0.001). Implant costs were higher for SBP when compared to PCR, whereas disposable equipment costs were higher for PCR when compared to SBP. The total mean true facility cost was $7658.00 for SBP, significantly higher than that of PCR at $3429.00 (*P *< 0.001).

Conclusions

Despite the substantial reduction in personnel costs seen with SBP, the true facility cost of SBP was significantly higher than that of PCR. As this novel technology is used more ubiquitously and its price is negotiated down, the cost savings seen in personnel and OR time will become more significant. Future prospective cost analyses should follow up on the changes in implant costs and account for potential anesthesia cost savings.

## Introduction

Massive irreparable rotator cuff tears are challenging and debilitating injuries with ambiguous treatment guidelines and approaches [1,2]. There exist several options for the treatment of such tears, and these include tendon transfers, superior capsular reconstruction, reverse shoulder arthroplasty, and partial rotator cuff repair (PCR) [1,3,4]. Each of these surgical options entails a set of advantages and limitations, and as such, treatment is often catered to the individual case [1,3,4]. However, in recent years, a novel therapeutic option was established, with great therapeutic potential for a select population of massive irreparable rotator cuff patients. The subacromial balloon is a device that is used to depress the humeral head away from the acromion, thereby relieving friction and impingement seen in rotator cuff deficiency [5]. The procedure initially involves diagnostic arthroscopy of the glenohumeral joint to confirm the absence of significant cartilage disease and the integrity of the subscapularis and the teres minor, as well as evaluate the feasibility of superior rotator cuff repair to restore anatomy [5,6]. The balloon is then inserted and inflated in the subacromial space, which helps restore the proper kinematics of the shoulder and increases the efficiency of the surrounding muscles in the joint, causing improvements in function [5-7]. A randomized clinical trial by Verma et al. compared the outcomes of subacromial balloon procedure to PCR for a specific population of massive irreparable rotator cuff tears and reported non-inferior outcomes in the subacromial balloon group, with superior scores on early function and range of motion (ROM) when compared to that of PCR [6]. 

Financial considerations often play a prominent role in assigning the treatment of choice for both the patient and the medical institutions [8,9]. It is important to assess the costs of a certain therapeutic modality as this helps establish its value and balances its costs with the advantages it entails. In the setting of massive rotator cuff tears, different procedures have different financial implications with respect to facility personnel allocations and procedural costs. Given the introduction of the novel subacromial balloon as a possible solution for many rotator cuff tear patients, it is pivotal to explore these differences to determine the financial implications of each intervention [6]. These differences may also impact clinical decision-making and patient management. To our knowledge, no prior study has investigated or compared procedural cost differences in personnel and supply allocation of different treatment options for massive irreparable rotator cuff tears. As such, the purpose of this study was to quantify the true facility cost difference between subacromial balloon placement (SBP) and PCR procedures while taking into account postoperative outcomes. PCR was utilized for comparison due to it being one of the most commonly used procedures for massive irreparable rotator cuff tears, and for it having a similar surgical profile to SBP (both arthroscopic, no use of graft, minimally invasive, etc.). This article was previously presented as a meeting poster at the 2023 American Academy of Orthopedic Surgeons (AAOS) Annual Meeting.

## Materials and methods

Study design

This study is a comparative procedural cost analysis. A retrospective chart review was conducted on a prospective cohort of patients with massive rotator cuff tears (>5 cm) randomized to PCR versus SBP between 2015 and 2018. Inclusion criteria included patients who had irreparable massive rotator cuff tears and had failed nonoperative treatment. In addition, only tears that were posterosuperior and did not involve the subscapularis muscle were included. Exclusion criteria included patients with reparable rotator cuff tears, evidence of severe glenohumeral osteoarthritis, deltoid palsy, subscapularis tear or insufficiency, and concurrent infection. Available information on demographic variables, medical comorbidities, and postoperative outcomes were recorded for all patients. The duration of surgical procedures and financial costs of each operation were analyzed and assessed.

Dataset collected

All randomized patients with complete data were included in our study. The generated dataset included age, gender, body mass index (BMI), Charlson comorbidity index (CCI), date of surgery, length of stay, preoperative active ROM examination, postoperative active ROM outcomes, time-care intervals, and facility costs. ROM was measured by the same surgeon using the same methodology during physical exams, both preoperatively and postoperatively.

Definitions

Examination of active ROM, both preoperatively and postoperatively, was conducted by measuring the degrees of forward elevation, abduction, and external rotation for each patient.

**Figure 1:**
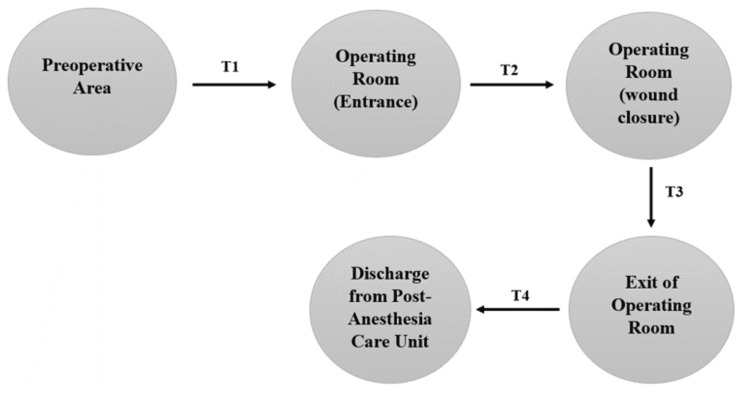
Distribution and definition of time-care intervals. Figure credit: All authors.

Time-care intervals about patient care were categorized into four durations and defined as follows: T1 was the interval spent in the preoperative area up until reaching the operating room (OR); T2 was the interval spent in the OR up until closure of the wound; T3 was the interval spent after wound closure up until exit of the OR; and finally, T4 was the interval spent after exit of the OR and up until discharge from postanesthesia care unit (Figure 1).

Facility costs were classified into personnel costs and supply costs. Personnel costs were calculated using a time-driven activity-based costing algorithm (TDABC) and were categorized according to the time intervals: T1 to T4. A study by Kaplan and Porter first introduced TDABC to measure facility costs based on the amount of time personnel needed [10]. The number and occupation of healthcare professionals associated with each time interval were retrieved. Afterward, an estimation of the average annual salary of each of these professionals was obtained from www.glassdoor.com, a renowned website that provides insight into the salaries and employment status of different institutions and companies (Table 1) [11]. A subsequent cost capacity rate (CCR), which reflects the cost per minute of an employee or space, was measured according to the employment schema of 50 weeks out of a year, five days per week, with eight hours per day of patient care. The same TDABC calculation method was used for both procedures. Supply costs were calculated by summation of the costs of all equipment, implants, and disposable devices used in the procedures during their designated time-care intervals. Total facility costs were subsequently calculated by adding the personnel costs and the supply costs of each procedure.

**Table 1:** Distribution of healthcare personnel salaries and cost per minute according to designated time intervals. (Surgeon fee varies according to surgical time.) CPM, cost per minute; PACU, post-anesthesia care unit; CRNA, certified registered nurse anesthetist

Healthcare personnel	Annual salary (USD)	Hours	Minutes	CPM (USD)
T1				
Preop nurse	75,509	2,000	120,000	0.63
Anesthesiologist	400,000	2,000	120,000	3.33
Total				3.96
T2				
Circulator nurse	72,502	2,000	120,000	0.60
Scrub nurse	47,599	2,000	120,000	0.40
Surgeon fee	-	-	-	-
FA	98,622	2,000	120,000	0.82
Anesthesiologist	400,000	2,000	120,000	3.33
CRNA	88,332	2,000	120,000	0.74
Total				5.89
T3				
Circulator nurse	72,502	2,000	120,000	0.60
Anesthesiologist	400,000	2,000	120,000	3.33
CRNA	88,332	2,000	120,000	0.74
Orderly × 2	32,341	2,000	120,000	0.54
Total				5.21
T4				
PACU nurse	75,509	2,000	120,000	0.63
Total				0.63

Procedures

Procedures compared in this study were arthroscopic PCR and arthroscopic SBP. All procedures were performed by the same fellowship-trained surgeon with more than 20 years of experience at a single surgical center. SBP involves introducing the subacromial balloon spacer, which is a biodegradable implant made out of poly L-lactide-co-ε-caprolactone, into the subacromial space. It is then filled with saline to help space out the acromiohumeral interval. None of the patients in the subacromial balloon group underwent concomitant partial cuff repair. Patients who had partial rotator cuff repair underwent suture anchor repair. Both procedures involved initial diagnostic arthroscopy of the glenohumeral joint.

Data analysis

Descriptive statistics were performed for all demographic variables. An independent t-test was used to explore for any statistical differences in the demographic information between the participant groups of each procedure. The independent t-test was also used to explore any statistical difference between the ROM measurements, time interval durations, personnel costs, and total facility costs between the procedures. All statistical analyses were performed using IBM SPSS Statistics for Windows, Version 25.0 (Released 2017, IBM Corp., Armonk, NY, USA), and an alpha level of 0.05 was used to evaluate statistical significance.

Ethical approval

The patient population included in our study was part of a prospective randomized controlled trial that had previously obtained Institutional Review Board (IRB) approval. A new amendment (#21E.333) was submitted to the IRB to conduct this study, which got approved.

## Results

Demographics

Our study population initially included 18 patients. Two patients were excluded due to incomplete information, and as a result, a total of 16 patients were included (*N *= 16): eight males and eight females. Seven underwent arthroscopic PCR, and nine underwent arthroscopic placement of a subacromial balloon. The mean age was 67.1 years for the repair group and 65.1 years for the balloon group. The percentage of female patients was 57.1% in the repair group and 44.4% in the balloon group. The mean BMI was 28.9 in the repair group and 28.7 in the balloon group. CCI was 0.57 in the repair group and 0.33 in the balloon group. And, finally, only one patient in the repair group spent a night in the hospital, constituting an average length of stay of 0.14 days, whereas none of the patients in the balloon group did. There was no statistical significance between any of the aforementioned variables between the two groups (Table 2).

**Table 2:** Demographic characteristics of patients undergoing partial cuff repair and subacromial balloon placement. PCR, partial cuff repair; SBP, subacromial balloon placement; LOS, length of stay; CCI, Charlson comorbidity index; BMI, body mass index

Demographics	PCR	SBP	*P*-value
N	7	9	
Age (years)	67.1	65.1	0.612
Gender (Females)	4 (57.1%)	4 (44.4%)	0.642
BMI (kg/m^2^)	28.9	28.7	0.944
CCI	0.57	0.33	0.472
LOS (days)	0.14	0	0.271

ROM outcomes

Preoperative ROM assessments were similar between the two groups, with no significant differences between any of the measurements. The average preoperative forward elevation was 111.4° in the repair group compared to 97.5° in the balloon group (*P *= 0.52). The average preoperative abduction was 77.1° in the repair group compared to 58.1° in the balloon group (*P *= 0.141). Finally, the average preoperative external rotation was 30.8° in the repair group, compared to 28.1° in the balloon group (Table 3).

On the other hand, about postoperative ROM assessments, a significant difference was obtained upon measurement of external rotation, which was 8° in the repair group and 36.9° in the balloon group (*P *= 0.023). No significant differences between the two groups were observed for postoperative forward elevation (*P *= 0.684) or postoperative abduction (*P *= 0.832) (Table 3).

**Table 3:** Preoperative and postoperative outcomes of patients undergoing partial cuff repair and subacromial balloon placement. ^*^P-value <= 0.05 is statistically significant. PCR, partial cuff repair; SBP, subacromial balloon placement

Range of motion	PCR, *n* (%)	SBP, *n* (%)	*P*-value
Preoperative measurements			
Forward elevation	111.4 (28.5)	97.5 (48.6)	0.520
Abduction	77.1 (21.4)	58.1 (25.1)	0.141
External rotation	30.8 (20.8)	28.1 (12.5)	0.766
Postoperative measurements			
Forward elevation	114.3 (30.3)	121.7 (38.5)	0.684
Abduction	74 (20.4)	77 (22.8)	0.832
External rotation	8.0 (17.9)	36.9 (20)	0.023*

Time intervals

Average T1 was 201.3 minutes in the repair group, higher than that of the balloon group at 117.9 minutes - albeit the difference did not reach significance on statistical analysis (*P *= 0.057). Similarly, there was no statistical significance between the two groups in average T3 duration (*P *= 0.292) and average T4 duration (*P *= 0.194). However, with respect to the T2 duration, the repair group recorded an average of 231 minutes, significantly greater than that of the balloon group at 102.8 minutes (*P *< 0.001) (Table 4).

**Table 4:** Duration (minutes) of the different time intervals associated with partial cuff repair and subacromial balloon placement procedures. ^*^P-value <= 0.05 is statistically significant. PCR, partial cuff repair; SBP, subacromial balloon placement

Time interval	Duration		*P*-value
	PCR (minutes)	SBP (minutes)	
T1	201.3	117.9	0.057
T2	231.3	102.8	<0.001*
T3	11.1	9.1	0.292
T4	74.3	60.1	0.194
Total time	518	289.9	<0.001*

Facility cost

The TDABC of T1 was $797.61 for PCR, higher than that of SBP, which was $467.14; nevertheless, that difference failed to reach significance (*P *= 0.057). On the other hand, the TDABC of T2 was $1362.76 for PCR, significantly higher than that of SBP, which was $605.58 (*P *< 0.001). No statistical difference was found between the two procedures for the TDABCs of T3 and T4. The total TDABC costs for all time intervals was $2,265.20 for the PCR, significantly higher than that of SBP at $1,158.04 (*P* < 0.001; Table 5).

**Table 5:** Associated costs (USD) of the different time intervals associated with partial cuff repair and subacromial balloon placement procedures. ^*^P-value <= 0.05 is statistically significant. PCR, partial cuff repair; SBP, subacromial balloon placement

Time interval cost (USD)			
	PCR	SBP	*P*-value
T1 cost	797.61	467.14	0.057
T2 cost	1362.76	605.58	<0.001*
T3 cost	58.08	47.49	0.292
T4 cost	46.74	37.82	0.194
Total cost	2,265.20	1,158.04	<0.001*

The average PCR procedure required the following equipment: 3.2 anchors, one cannula, and one burr. On the other hand, the average SBP procedure required a subacromial balloon implant. Nevertheless, and according to the prices presented in (Table 6), that would constitute a supply cost of $6,500 for the subacromial balloon procedure higher than that of PCR, whose supply cost was $1,164. Accordingly, the total facility cost was $7,658 for SBP, significantly higher than that of the partial cuff pair at $3,429 (*P *< 0.001).

**Table 6:** Supply costs associated with partial cuff repair and subacromial balloon placement procedures. PCR, partial cuff repair; SBP, subacromial balloon placement

Supply usage	PCR	SBP
Supply count	3.2 anchors	1 balloon
Supply cost	$944.00	$6,500.00
Disposables count	1 cannula, 1 burr	-
Disposables cost	$70.00, $150.00	-

## Discussion

Our study assessed the facility cost differences between two procedures used to treat massive irreparable rotator cuff tears: arthroscopic PCR and SBP. SBP proved to have significantly better active ROM outcomes when compared to repair, specifically in postoperative external rotation. In addition, the SBP procedure had a significantly shorter time of care, given the prominently decreased operating time. Nevertheless, total facility costs remain higher for SBP when compared to PCR, given the novelty of this procedure in shoulder surgery and the high cost of the implanted device.

SBP is a new innovative treatment option in the setting of massive rotator cuff tears, having emerged in the early 2010s [6,12]. It is a procedure where a biodegradable balloon is arthroscopically inserted into the glenohumeral joint in patients with superior rotator cuff deficiency, thereby increasing the distance between the humeral head and the acromion [6]. It is an effective treatment strategy for a specific subset of shoulder patients: those with a massive superior rotator cuff tear, normal deltoid function, minimal or mild arthritis, and intact subscapularis and teres minor muscles [6,7,12,13]. The balloon decreases pain by alleviating the impingement of the superiorly migrating humeral head on the acromion and improves function by altering the biomechanics of the shoulder in a way that increases the efficiency of adjacent muscles in the shoulder [6,7]. This allows these muscles to compensate for the deficient superior rotator cuff and helps to restore proper joint kinematics through optimization of humeral head position and the compressive force coupling of the subscapularis and the teres minor [6,7]. SBP can help such patients in a way that is comparable to other treatment options, as demonstrated by our results, and by others published in the literature [6,12,14]. The randomized controlled trial by Verma et al. demonstrated similar outcomes for both PCR and SBP for patients with massive irreparable rotator cuff tears at two years follow-ups [6]. A retrospective study by Kaisidis et al. explored 47 patients who underwent SBP for massive irreparable rotator cuff tears and reported significant improvements in the constant score and visual analog scale (VAS) score at two years follow-ups [12]. Another systematic review by Davies et al. evaluated the different treatment options available for the management of massive irreparable rotator cuff tears and demonstrated similarly improved outcomes between arthroscopic PCR, SBP, superior capsular reconstruction, and arthroscopic debridement at short-term follow-up [14].

In addition to advantages with respect to outcomes, the SBP requires minimal surgical time when compared to other arthroscopic procedures [15-17]. Once the physician confirms that the subacromial balloon is the best option for this patient, the proper implant size was selected and prepared, and the balloon was arthroscopically inserted into the subacromial space and filled with saline to fully expand it [7]. The average T2 recorded in our study for SBP was around 102 minutes, including the time needed to anesthetize and prepare the patient, as well as the closure of the surgical wounds. Studies have shown that the time required for the procedural placement of the balloon ranges between 2 and 30 minutes, with a few studies reporting an average of 10 minutes [15-17]. Accordingly, the balloon offers significantly reduced surgical times when compared to other procedures like PCR [18]. This reduced time entails multiple benefits as it decreases the risks associated with prolonged anesthesia to the patient and implies financial and surgical time cost advantages to medical and surgical institutions [19,20].

Tackling cost differences is very important when comparing different surgical procedures [19]. Our study demonstrated that the reduced surgical time in the SBP translated into significantly reduced TDABCs when compared to PCR. In addition, the cost of disposable equipment was higher in the PCR when compared to SBP. Nevertheless, the total facility costs remained significantly higher for the SBP procedure when compared to the PCR, mainly due to the high price of the balloon implant - the balloon implant constituted more than 80% of the total facility costs of the SBP procedure. On the other hand, the implanted devices used for the PCR in our study, which included on average 3.2 anchors, accounted for around 42% of the total cost only.

The novelty of the subacromial balloon sets limits on the ability to decrease implant costs, as production may be confined to a few medical device companies worldwide. As such, there is not much ability, at this time, to negotiate lower production costs that may result in subsequently lower supply costs. With time, however, the ubiquitous use of this procedure may allow for competitive bidding by production facilities, and this can help prominently decrease the commercial cost of the balloon implant [21]. Hence, the SBP has the potential to be one of the most cost-effective procedures for the management of rotator cuff pathology in the future. Along these lines, a study by Castagna et al. showed that the balloon was the most cost-effective treatment with respect to quality-adjusted life years when compared to other relevant treatment options [18]. Additional cost analysis studies are required in the future to keep up with the developments of this technology, monitor its concurrent production costs, and address its time savings about decreased surgical time (as the learning curve improves) and reduced anesthesia cost savings.

Our study is one of the first to explore the cost efficiency of the SBP in comparison to other rotator cuff procedures. Nevertheless, several limitations exist. The small population in our study may predispose the results to cognitive bias and can impact the reliability of the study. In addition, we were limited by the retrospective nature of our study design, and as such, exploring additional cost variables and patient-reported outcomes was not possible. That being said, this study remains impactful and valuable to the literature, as it offers insight into the financial implications of a novel technology and compares it to other available treatment options using a randomized population of patients.

## Conclusions

Different options exist for the treatment of massive irreparable rotator cuff tears, with each providing a certain set of advantages and disadvantages for a population of suitable patients. The subacromial balloon is a new treatment option that provides improvements in pain, function, and ROM for patients with specific indications. The minimal surgical time and the lack of disposable equipment needed during the surgery allow it to be a very cost-efficient procedure when compared to other operations. Nevertheless, the cost of the implanted balloon device remains high due to the novelty of this technology and the high production prices. As a result, our study showed that despite the cost savings provided by the decreased surgical time and lack of disposables, the procedure was burdened by high implant costs. As the SBP procedure becomes more ubiquitous, lower production prices can be negotiated, and the facility costs would subsequently decrease, leading it to become a more cost-effective treatment option for patients with massive irreparable rotator cuff tears. That being said, future studies exploring the financial implications of this surgery are necessary to determine its true cost efficiency when compared to other surgical options.
